# An Exosome-Laden Hydrogel Wound Dressing That Can Be Point-of-Need Manufactured in Austere and Operational Environments

**DOI:** 10.3390/bioengineering11080804

**Published:** 2024-08-08

**Authors:** E. Cate Wisdom, Andrew Lamont, Hannah Martinez, Michael Rockovich, Woojin Lee, Kristin H. Gilchrist, Vincent B. Ho, George J. Klarmann

**Affiliations:** 1 USU Center for Biotechnology (4DBio3), Department of Radiology, Uniformed Services University of the Health Sciences, 4301 Jones Bridge Road, Bethesda, MD 20814, USAkristin.gilchrist.ctr@usuhs.edu (K.H.G.); vincent.ho@usuhs.edu (V.B.H.); george.klarmann.ctr@usuhs.edu (G.J.K.); 2 The Geneva Foundation, 917 Pacific Ave, Tacoma, WA 98402, USA; 3 The United States Air Force Academy, 2304 Cadet Drive, USAF Academy, CO 80840, USA; 4 School of Medicine, Uniformed Service University of the Health Sciences, 4301 Jones Bridge Road, Bethesda, MD 20814, USA; 5 The United States Naval Academy, 121 Blake Rd., Annapolis, MD 21402, USA; 6 The United States Military Academy, 606 Thayer Rd., West Point, NY 10996, USA

**Keywords:** exosomes, additive manufacturing, point-of-need manufacturing, austere environment, alginate, bioprinting, wound healing

## Abstract

Skin wounds often form scar tissue during healing. Early intervention with tissue-engineered materials and cell therapies may promote scar-free healing. Exosomes and extracellular vesicles (EV) secreted by mesenchymal stromal cells (MSC) are believed to have high regenerative capacity. EV bioactivity is preserved after lyophilization and storage to enable use in remote and typically resource-constrained environments. We developed a bioprinted bandage containing reconstituted EVs that can be fabricated at the point-of-need. An alginate/carboxymethyl cellulose (CMC) biomaterial ink was prepared, and printability and mechanical properties were assessed with rheology and compression testing. Three-dimensional printed constructs were evaluated for Young’s modulus relative to infill density and crosslinking to yield material with stiffness suitable for use as a wound dressing. We purified EVs from human MSC-conditioned media and characterized them with nanoparticle tracking analysis and mass spectroscopy, which gave a peak size of 118 nm and identification of known EV proteins. Fluorescently labeled EVs were mixed to form bio-ink and bioprinted to characterize EV release. EV bandages were bioprinted on both a commercial laboratory bioprinter and a custom ruggedized 3D printer with bioprinting capabilities, and lyophilized EVs, biomaterial ink, and thermoplastic filament were deployed to an austere Arctic environment and bioprinted. This work demonstrates that EVs can be bioprinted with an alginate/CMC hydrogel and released over time when in contact with a skin-like substitute. The technology is suitable for operational medical applications, notably in resource-limited locations, including large-scale natural disasters, humanitarian crises, and combat zones.

## 1. Introduction

Austere and operational environments present unique challenges for early and prolonged treatment of wounds resulting from burns, blasts, gunshots, or other traumas. While improvements in operational medicine, as well as safety measures, have increased the survival of injured patients, the overall number of extremity injuries has risen during the most recent military conflicts [1]. The average time for a burn-injured American service member to reach definitive care was three to four days during Operation Iraqi Freedom and Operation Enduring Freedom [2]. The evacuation time for injured service members will likely be much longer in future conflicts where multi-domain operations against near-peer adversaries are expected [2]. Additionally, contemporary conflicts such as those in Syria, Yemen, and Libya are characterized by fighting in city centers with high civilian populations, increasing the probability of overwhelming local medical infrastructure, which is likely sparsely resourced. Medical care for civilians during conflict when the local health system breaks down is uncoordinated between local hospitals and international humanitarian non-governmental organizations [3]. Similarly, following large-scale natural disasters, hospital infrastructure becomes overwhelmed, support from international aid can be uncoordinated, and evacuation limited or not feasible. In these scenarios, advanced therapeutic options by responding healthcare workers in forward austere locations may be required.

Treatment of wounds early in forward locations would benefit from advanced tissue engineering approaches to promote scar-free healing [4,5,6]. In addition to the superficial cosmetic consequences, scar tissue can cause significant physiologic constraints such as limiting the range of motion across joints, interrupting normal fascial planes, complicating normal physiologic motion of internal structures such as the bowel, and complicating or even precluding surgical revisions and reconstructions of complex injuries. Tissue engineering strategies, including biomaterial and cell-based therapies, are promising for significantly improving wound closure, collagen synthesis, and vessel formation [7].

Alginate is a naturally occurring anionic biomaterial derived from brown seaweed or algae that is low cost, crosslinks with divalent calcium ions, and is biocompatible [8,9,10]. Alginate wound dressings are available commercially and are used to treat acute wounds [11]. Additionally, alginate dressings maintain a moist environment needed for wound healing and reduce bacterial infections [12]. Carboxymethyl cellulose (CMC) is an anionic derivative of cellulose that has been widely used in tissue engineering [13]. Anionic cellulose materials have been used in wound dressings due to their ability to promote wound healing by modulating matrix metalloproteases (MMP) [11]. This is achieved by the negatively charged cellulose material dislodging the positively charged metal ions from the MMPs and destroying the structure of these enzymes, rendering them inactive [11]. Alginate/CMC wound dressings are known to shorten wound healing time and help prevent progression to infection [14].

Mesenchymal stromal cells (MSC) have shown greatly improved wound healing with accelerated wound closure, increased re-epithelialization, cellularity, and angiogenesis [15]. Due to the requirements of cold-chain logistics and dedicated laboratory culture space, MSCs would be difficult to incorporate into point-of-need wound dressings. However, MSC-derived exosomes and extracellular vesicles (EV) retain some of the key wound healing activities associated with the parental MSC, including immunomodulatory properties and high regenerative capacity [16]. EV bioactivity is preserved following lyophilization and storage [17,18], which could enable use in austere, resource-constrained environments. The application of EV-based therapies at the point-of-need presents an opportunity to bring the benefits of modern tissue engineering and cell-based strategies to resource-limited environments.

Point-of-need manufacturing of wound dressings that incorporate these advanced therapies could improve wound healing and treatment of injured patients during conflict or crisis where an increased number of injuries can overwhelm existing medical infrastructure, and evacuations are difficult. We previously demonstrated that a ruggedized 3D printer could be transported to and successfully operated in austere, Arctic, and desert environments [19,20]. Three-dimensional printing may help the logistical challenges of health care in austere environments and can be used to fabricate objects from materials ranging from hydrogels to thermoplastics. The 3D prints are guided by digital models that can be easily adapted to provide a bandage of customizable size and shape. For example, wounds near or involving the airway could benefit from a rigid backing to protect the trachea while promoting contact of bioactive components with the wound. 3D printing enables patient-specific, customizable bandages to facilitate these types of medical needs [21]. 3D bioprinting has the potential to bring advanced therapeutics to traditional thermoplastic prints by incorporating bioactive materials into hydrogels and pastes.

In this work, we developed a bioactive bandage using a custom alginate/CMC bio-ink laden with EVs. First, the printability and mechanical properties of the alginate/CMC biomaterial ink were assessed. Then, we incorporated lyophilized EVs and characterized their release from the dressing into a collagen skin substitute. Finally, we translated the fabrication of the bioprinted bandage from the commercial laboratory 3D bioprinter to the ruggedized 3D printer, and we demonstrated the feasibility of incorporating EVs into an alginate/cellulose bio-ink in an austere Arctic environment. This work demonstrates an example of the point-of-need manufacturing of advanced therapeutics in austere and operational medical environments.

## 2. Materials and Methods

### 2.1. Exosome Isolation and Lyophilization

EVs were isolated using size exclusion chromatography (SEC) from human bone marrow-derived mesenchymal stem/stromal cell (hBMSC) conditioned media (RoosterBio, Federick, MD, USA). The cell-conditioned media (CCM) was first cleared of large debris by filtering with a 0.22 µm syringe filter (Millipore Sigma, Burlington, MA, USA). The filtered CCM was concentrated using a 100-kDA molecular weight cut-off Amicon centrifugal filter (Millipore Sigma, Burlington, MA, USA). One milliliter of concentrated CCM was loaded onto a 35 nm series QEV2 isolation column (Izon, Medford, MA, USA) that had been preconditioned by exchanging 1.5 volumes of phosphate-buffered saline (PBS). Next, 3.0 mL of PBS was added to the column. Once the PBS had eluted out of the column, the reservoir was filled with PBS, and the EV fraction was collected. All flow was gravity-assisted.

Isolated EVs were lyophilized in a solution containing 50 mM trehalose in PBS lyoprotectant (Sigma–Aldrich, St. Louis, MO, USA) [22]. The solution containing EVs in microcentrifuge tubes was frozen at −80 °C overnight. Prior to placement on the lyophilizer, centrifuge tubes were opened and covered with parafilm. Three vent holes were punched in the parafilm using a 16-gauge needle, then the vented tubes were loaded into a 2.5 L −80 °C Benchtop Lyophilizer (Labconco Corporation, Kansas City, MO, USA) and held at 0.1 mBar for 24 h.

### 2.2. Exosome Characterization

The count and size distribution of EVs collected were measured using nanoparticle tracking analysis (NTA, Nanosight NS300, Malvern, Westborough, MA, USA). NTA analysis was conducted on samples of at least 10 particles per frame. Five runs of 60 s duration and flowrate of 4 µL per minute were averaged to determine the particle size distribution and concentration of particles in the samples.

### 2.3. Transmission Electron Microscopy

EV samples for transmission electron microscopy (TEM) were initially fixed in 4% paraformaldehyde in PBS for 1 h. For lyophilized samples, EVs were first hydrated in PBS. Next, the fixed EV samples were applied to a carbon Formvar film-coated TEM grid and incubated at room temperature for 20 min. Following incubation, the samples were wicked with a Kimwipe, washed with PBS, fixed with 1% glutaraldehyde for 5 min, washed with water, and stained with 1% uranyl acetate for 30 s. Following staining, the remaining liquid was wicked off with a Kimwipe, and the samples were allowed to dry. A JEOL-2100F TEM (JEOL, Peabody, MA, USA) at the University of Maryland NanoCenter was used to image samples. Samples were imaged at 110 kV with an EELS spectral camera.

### 2.4. Mass Spectroscopy of Exosomes

EVs were analyzed by LC/ESI-TOF mass spectrometry at System Biosciences (Palo Alto, CA, USA). Proteins were lysed and separated with gel electrophoresis and then trypsinized. Peptide sequences were compared to known protein sequences as a service provided by the company. The false discovery rate (FDR) threshold for high-confidence sequence determination and protein identification was <0.01.

### 2.5. Exosome Staining

EV lipid bilayers were labeled with the lipophilic fluorophore PKH26 (Sigma–Aldrich, St. Louis, MO, USA). Isolated EVs in PBS at a concentration of 0.5 µg/µL were stained with 8 µM PKH26 in diluent C and incubated for 5 min. Following incubation, the excess stain was bound with fetal bovine serum (Sigma–Aldrich, St. Louis, MO, USA). The EVs were then purified from unbound stain by isolation with SEC. Studies evaluating EV transfer from the alginate/CMC dressing to the skin substitute were conducted with 400 µg PKH26 red fluorescently labeled EVs. The labeled EVs were mixed in the alginate/CMC biomaterial ink at a 1:5 ratio (*v*/*v*) and bioprinted using the same parameters as the alginate/CMC dressing.

### 2.6. Alginate Cellulose Biomaterial Ink Formulation

The biomaterial ink chosen was composed of alginate and CMC [23]. Biomaterial ink was made in batches of 100 mL in a base of 15% PBS and contained 3.33 g of medium viscosity alginate (A2033, Sigma–Aldrich, St. Louis, MO, USA), 4 g of medium viscosity carboxymethyl cellulose (150560, MP Biochemicals, Solon, OH, USA), 2 g low viscosity alginate (BM25266.30, Thermo Scientific, Frederick, MD, USA), 0.1 g of CaCl_2_ (793639, Sigma–Aldrich, St. Louis, MO, USA) and 0.1 g of CaSO_4_ (C3771, Sigma–Aldrich, St. Louis, MO, USA). Dry powders were premixed and then slowly added to the liquid while vigorously stirring. When increasing viscosity halted stir bar movement, the biomaterial ink was heated to 65 °C, and the remaining powder was added over the course of 2 h. Finally, the mixture was autoclaved and stored at room temperature until use.

### 2.7. Biomaterial Ink Characterization

The rheological properties of the biomaterial ink were evaluated with a rheometer (MCR302, Anton Parr, Graz, Austria) equipped with a parallel plate (PP25) and controlled with instrument software (RheoCompass v1.24). An oscillatory shear amplitude sweep from 0.05% to 100% shear strain at 1 Hz and 23 °C with a 0.5 mm gap was used to determine the linear viscoelastic range for the biomaterial ink. Viscosity was monitored at three different temperatures where the shear rate was varied from 0.005 to 200 s^−1^ in a ramp logarithmic profile with 1 Hz frequency and a 0.5 mm gap. A controlled shear rate test was performed at 23 °C using a 0.5 mm gap with shear stress varied in a ramp linear manner from 1 Pa to 300 Pa and used to extract the yield stress point (inflection point) using a calculation performed by instrument software.

### 2.8. Bioactive Dressing Printing on a Commercial Laboratory Bioprinter

A commercial bioprinter (BioX; Cellink, Gothenburg, Sweden) was used to print the dressings. Biomaterial ink was loaded into 3 mL syringes attached to 22-gauge (0.4 mm) conical-tipped needles (Nordson EFD, Westlake, OH, USA). Speed was held constant at 6 mm/sec, and pressure (80–100 kPa) was varied until extrusion shapes and biomaterial ink volumes closely matched the calculated object volume. Following 3D bioprinting, the objects were crosslinked with 3.0 mL of a 2% solution of CaCl_2_ (Sigma–Aldrich, St. Louis, MO, USA) in water various times in a 6-well plate and then stored in PBS as necessary. Printed dressings were generally used within several hours post-crosslinking.

### 2.9. Mechanical Testing

3D bioprinted and crosslinked dressings were compressed on a compression tester (UniVert, CellScale Biomaterials Testing, Waterloo, ON, Canada) equipped with load cells ranging from 2.5 N to 10 N. All samples were stored in PBS for at least 1 h prior to testing. Samples with residual PBS removed were placed on the flat plate for unconfined compression testing. Compression strains up to 10% were applied over a 30 s period. Force versus displacement data were collected by the instrument software (UniVert, version 1.0.0.1 CellScale Biomaterials Testing, Waterloo, ON, Canada). Stress was calculated by dividing the force by surface area, and the strain was calculated by taking the distance moved by the upper plate and dividing it by the initial height of the bioprinted dressing material. Stress versus strain values were plotted, and the curve from the initial stages of compression was fit to a linear regression where the resulting slope value was Young’s modulus. The linearity of the curve fit was maintained by limiting the data range to ensure *R*^2^ values were above 0.98.

### 2.10. EV Release from Bioprinted Dressing

Bioprinted dressings made with EV-laden alginate/CMC bio-ink were cut into 10 mm × 10 mm × 3 mm sizes after crosslinking and placed atop 10 mm × 10 mm × 3 mm printed collagen squares. Collagen squares were 3D bioprinted with collagen methacrylate (Advanced Biomatrix, Carlsbad, CA, USA) and lithium phenyl-2,4,6-trimethylbenzoylphosphinate (LAP) photoinitiator into a sheared agarose support bath, and crosslinked with 405 nm light using methods previously described [24]. The stacked dressings on the collagen squares were incubated at 37 °C with 1 mL PBS in a 6-well plate to keep the collagen squares moist. At various time points, the collagen square was analyzed by confocal microscopy (Stellaris, Leica, Deerfield, IL, USA) for the presence of fluorescent EVs that resulted from transfer from the alginate/CMC dressing.

### 2.11. Logistics and Transportation of Materials for Austere Bioprinting Exercise

A ruggedized 3D printer with bioprinting capabilities (nScrypt, Orlando, FL, USA), polylactic acid (PLA) filament (Dremel, Mt. Prospect, IL, USA), alginate/cellulose biomaterial ink, and lyophilized EVs were transported to a rural Arctic locale. The materials and equipment were carefully packaged in a wooden pallet crate (ULINE, Pleasant Prairie, WI, USA) and shipped using a commercial carrier (DHL) [20]. The pallet was received and repackaged in a rental car for transportation to the test site.

### 2.12. Bioactive Bandage Printing on a Ruggedized 3D Printer

Computer-aided design (CAD) files for a bandage model printed on a ruggedized printer in an austere desert environment were used to improve upon and evaluate the effort in an Arctic environment [19]. The bandage consisted of a PLA backing, alginate/cellulose bio-ink loaded with EVs, and a biocompatible acrylic adhesive (Duo Lash Adhesive Clear, Ardell Inc. Redmond, WA, USA). The PLA backing was 3D printed with an extrusion filament print head (nFD, nScrypt, Orlando, FL, USA). The adhesive was transferred into a 3 mL syringe barrel (Nordson EFD, Westlake, OH, USA) and loaded onto a pneumatic microvalve printhead (SmartPump, nScript, Orlando, FL, USA). A second pneumatic microvalve printhead was used to bioprint the alginate/cellulose bio-ink loaded with reconstituted EVs. A total of 50 µg of the lyophilized EVs were reconstituted in 100 µL PBS and mixed with 2 mL of bio-ink. Mixing was achieved by pipetting the reconstituted EVs into a 3 mL syringe barrel containing 2 mL of bio-ink. The alginate/cellulose bio-ink with EVs was mixed by connecting a syringe using a luer adaptor to the syringe barrel and gently mixing the bio-ink back and forth. Once mixed, the bio-ink was completely transferred to the syringe barrel and loaded onto a pneumatic microvalve printhead.

The bandage CAD files, including the PLA backing, hydrogel dressing, and adhesive, were uploaded into the ruggedized printer software (MTGen 3.7.6 software, nScrypt, Orlando, FL, USA). The CAD files were arranged in the software and sliced. Three-dimensional print parameters for each component of the bandage were input. For the PLA backing, a 450 µm ceramic tip was used with a nozzle temperature of 210 °C and a print bed temperature of 50 °C. The adhesive and alginate/cellulose hydrogel with EVs were bioprinted using 125 µm ceramic tips at 6 kPa and 8 kPa pressure at a speed of 20 mm/s and 10 mm/s, respectively.

## 3. Results and Discussion

### 3.1. Exosome Isolation, Lyophilization and Characterization

**Figure 1:**
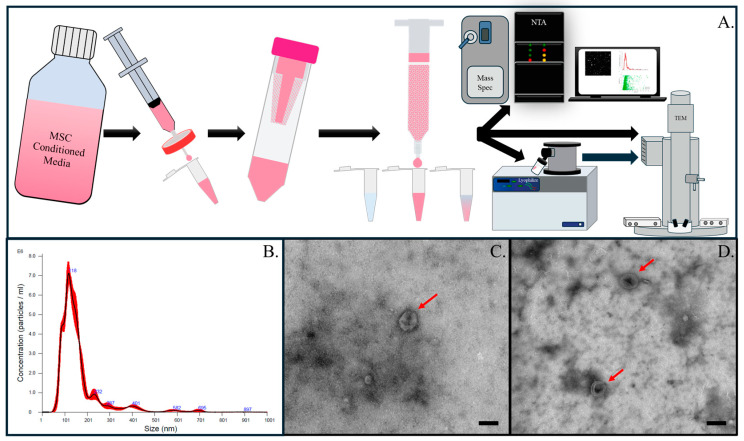
Isolation and characterization of EVs from MSC-conditioned media. (**A**) Schematic of EV isolation using size exclusion chromatography followed by lyophilization and characterization using mass spectrometry, NTA, and TEM (**B**). NTA of EVs collected showed a particle size peak of 118 nm with a concentration of 6.67 × 10^9^ +/− 1.69 × 10^7^ EVs/mL. Fresh EVs (**C**) and EVs following lyophilization (**D**) were imaged with TEM. Red arrows are pointing to the fresh (**C**) and lyophilized EVs (**D**) imaged with TEM.

EVs from hBMSCs were isolated using SEC (Figure 1). SEC has been shown to better preserve protein expression compared to ultracentrifugation [25,26]. The exosome fraction of the column flow-through was determined by measuring the absorbance at 280 nm of a small volume (~2 µL) sample for each isolated fraction (Supplementary Information Figure S1). This fraction was further evaluated to confirm the isolation of EVs using NTA and mass spectrometry. NTA of isolated exosomes showed a particle size peak at 118 nm (Figure 1B), which is consistent with literature-reported values of EV size [27]. Following isolation, EVs were preserved by lyophilization with a 50 mM trehalose lyoprotectant according to a previously reported protocol [22]. EVs were lyophilized since it was shown that freeze-dried EVs retain function [28,29] and have storage stability [28,30]. Maintaining function and having storage stability are both important factors in operational or austere environments where cold storage is not always available. TEM micrographs show the presence of EVs in both fresh and reconstituted lyophilized samples (Figure 1C,D).

The isolated EVs were analyzed with mass spectrometry and showed the presence of known EV group 1 proteins CD63 (TSPAN30), CD90 (THY1) integrin subunit beta, and syndecans-1 and 4; known EV group 2 proteins annexin A, HSPA8 (heat shock cognate 71 kDa protein), ALIX (PDCD6IP); and group 4 non-EV co-isolated materials which include histone proteins, (Table 1) [31]. Group 3 proteins are used to measure levels of EV purity, and except for ribosomal proteins, other common contaminants like apolipoproteins are present in amounts too low to quantify. These data demonstrate that EVs were successfully isolated from MSC-conditioned growth media.

### 3.2. Development of an Alginate/CMC Biomaterial Ink

Alginate biomaterial ink is known for biocompatibility, mild crosslinking with divalent (CaCl_2_) ions, 3D printability, and tunable pore size (5–200 nm), which facilitates the release of incorporated bioactive molecules [12,32]. The structure of alginate contains repeating blocks of D-mannuronate (M) and a-L-guluronate (G) residues that ionically crosslink by forming egg-box structures in the presence of divalent ions [33]. The organization, composition, and length of each block varies by the source of alginate [12]. An ideal bioactive wound dressing bio-ink would leverage the benefits of alginate mentioned previously but also support EV release. The use of other hydrogels, such as chitosan, has proven successful in 3D printed wound dressings [34,35]. While chitosan is antimicrobial [36,37] and biocompatible [38,39], it requires acidic conditions for solubilization, and EVs stored at pH 4.0 show decreased recovery relative to neutral pH [40]. Therefore, it was not selected for use in the EV-containing wound dressings examined in the work.

Alginate alone does not have sufficient biomaterial ink properties to support 3D printing. CMC is a biocompatible, water-soluble thickening agent commonly used as a food and drug additive. With regards to biomaterial inks like alginate, CMC can be added to improve the viscosity and 3D printability of hydrogel biomaterial inks [41]. However, others may wish to explore the use of other biocompatible anti-inflammatory viscosity modifiers, such as k-carrageenan [42,43].

**Figure 2:**
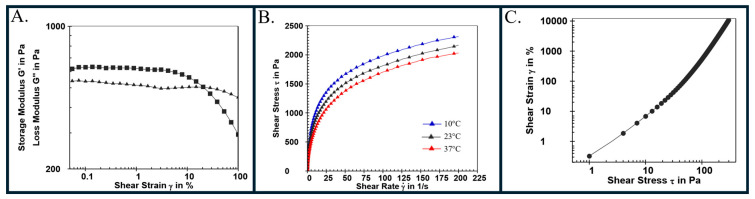
Rheology of alginate/CMC dressing biomaterial ink. A 0.05–100% shear strain sweep was performed at 23 °C on a parallel plate rheometer with a 0.5 mm gap and 1 Hz frequency (**A**). Storage modulus, G′ (black squares) and loss modulus, G″ (black triangles) and plotted. The storage modulus value was independent of shear strain up to approximately 1%, and G′ is greater than G″ indicating the biomaterial ink is a viscoelastic solid. (**B**) Viscosity study where biomaterial ink was loaded on a parallel plate rheometer with a 0.5 mm gap, 1 Hz frequency, and a ramp logarithmic program for shear rate was used from 0.005 to 200 s^−1^. The shear rate is plotted versus shear stress at 10 °C (blue triangles), 23 °C (black triangles), and 37 °C (red triangles). Increasing temperature decreased the viscosity. The biomaterial ink is non-Newtonian and shear thinning at each temperature. (**C**) Biomaterial ink yield stress determination at 23 °C. Samples were loaded on a parallel plate rheometer with a 0.5 mm gap. Shear stress was varied from 1 to 300 Pa using a ramp linear program. The yield stress was calculated using rheometer software.

In this work, a two-material hydrogel biomaterial ink was selected composed of alginate and CMC [23,44]. These materials, in combination, are known to have anti-infective properties [44], and both materials are generally regarded as safe by the US Food and Drug Association (FDA) [45]. Alginate/CMC biomaterial ink containing 5.3% alginate (2% low viscosity plus 3.3% high viscosity) and 4% CMC was developed and evaluated for printability using rheology. For rheological testing, an amplitude sweep from 0.05% to 100% strain at 1 Hz was used to determine the viscoelastic properties of the biomaterial ink at 23 °C and to define parameters to guide further rheological testing (Figure 2A). The measured elastic energy storage modulus (G′) value of 625 Pa is greater than the 520 Pa value of the loss (viscous) modulus (G′′), suggesting that the biomaterial ink is printable [46] and behaves like a viscoelastic solid. The linear viscoelastic region (LVE) was detected at shear strains from approximately 0.1% to 1%, where the G′ values are essentially constant and independent of the changing shear, and the relationship between the applied stress and the measured outputs is linear. At shear strains between 10% and 100%, the G′ value decreased and intersected the G′′ values at 20% shear strain, indicating shear-induced breakdown of the biomaterial ink is occurring. The storage and loss moduli are significantly less than a similar hydrogel biomaterial ink that was more extensively crosslinked with higher concentrations of Ca^2+^ ions prior to analysis and printing [44]. Though increased stiffness associated with pre-crosslinking of the alginate improves print shape fidelity, post-print crosslinking allows for more flexibility in tuning the stiffness, where needs can vary depending on the functionality required.

Biomaterial ink viscosity was measured at three potential printing temperatures (10 °C, 23 °C and 37 °C). A shear rate sweep from 0.005 to 200 s^−1^ was conducted, and shear rate vs. shear stress was plotted (Figure 2B). Viscosity was highest at 10 °C and lowest at 37 °C, and the biomaterial ink behaves as a shear thinning non-Newtonian fluid (pseudoplastic) at each temperature as demonstrated by the non-linear response of shear stress to increasing shear rate. Biomaterial ink must exhibit shear thinning characteristics to be printable where viscosity decreases as shear rate increases which allows biomaterial ink to flow under pressure. A printing temperature of 23 °C was used for the 3D bioprinting for the remainder of the work presented here. A controlled shear rate test was performed where shear stress (τ) was varied from 1 to 300 Pa to measure shear strain as a function of the applied shear stress (Figure 2C). The inflection point on the graph is the yield stress, which, for this biomaterial ink at 23 °C, was 54.8 ± 4.9 Pa. The yield stress is the minimal applied stress needed to overcome the gel-like structure of the biomaterial ink and initiate biomaterial ink flow and extrusion. The high measured yield stress indicates that deposited filaments should not deform too much after extrusion and not require a fluid gel support bath to maintain good overall printed shape fidelity as typically exhibited by collagen hydrogels [24]. Taken together, the rheology data indicates the alginate/CMC biomaterial ink is shear thinning and printable.

### 3.3. Mechanical Evaluation of Printed Dressings

Mechanical properties of the biomaterial ink were evaluated to ensure printability and compatibility for use as a wound dressing. Rheology was used to determine the flow characteristics of the biomaterial ink including the ability for the ink to be extruded using a pneumatic printhead and then remain in place once the shear stress from the printhead was removed. Compression testing was used to determine the modulus of the resulting hydrogel dressing with different crosslinking parameters. The modulus must be less than the modulus of human skin to provide comfort and prevent secondary trauma [47,48].

**Figure 3:**
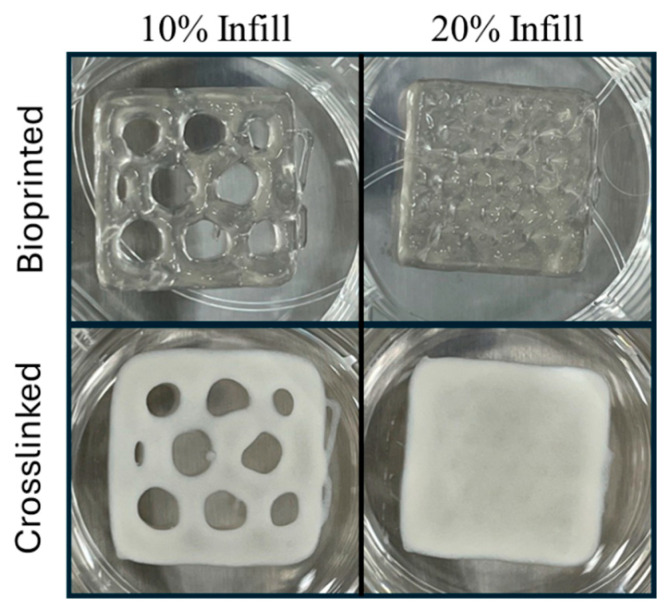
Print fidelity of alginate/CMC biomaterial ink at two different infill percentages before and after crosslinking with CaCl_2_. The bio-ink was 3D printed into a 20 × 20 × 3 mm object using a commercial bioprinter (BioX, Cellink). Print parameters were 6 mm/sec and up to 100 kPa pressure using a 22-gauge conical tip (**top row**). Infill was either 20% or 10%. Following printing, the prints were incubated in CaCl_2_ for 60 min to crosslink the alginate component (**bottom row**).

Biomaterial ink was evaluated for rheological properties to determine if the material was printable. Biomaterial ink was extruded into shapes of 20 × 20 × 3 mm structures using a 22-gauge conical tip, a speed of 6 mm/sec, and pressures ranging from 90 to 100 kPa using a commercial research grade bioprinter. The biomaterial ink extruded well, and print shape fidelity was maintained (Figure 3, top). Before crosslinking, the printed objects had the same outer dimensions as the CAD file. The fine detail resolution of the holes within the honeycomb pattern showed that internal print fidelity was not as good as the exterior dimensions. This may be the result of the biomaterial ink viscosity and yield stress and slight over-extrusion of the biomaterial ink, which is difficult to control using pneumatic, non-screw-driven print heads [24]. Following alginate crosslinking with CaCl_2_ (Figure 3, bottom), the outer dimensions measured 21.6 ± 0.3 mm × 21.7 ± 0.3 mm.

**Figure 4:**
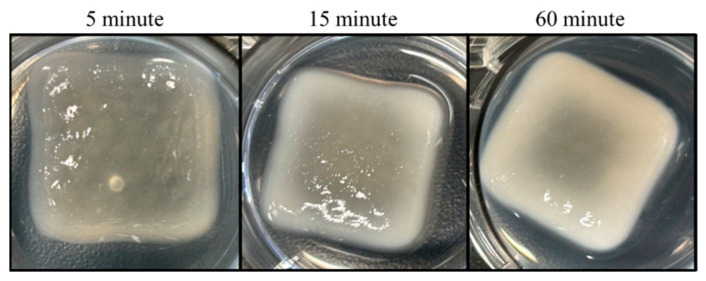
Printed Alginate/CMC hydrogel dressings with varying crosslinking times. Hydrogel dressings were printed with 20% infill and crosslinked in CaCl_2_ for 5, 15, or 60 min.

The prints were crosslinked for 5, 15, or 60 min. The alginate/CMC biomaterial ink changed color and became progressively more opaque with increasing crosslinking times (Figure 4). The biomaterial ink likely became more opaque due to increased crosslinking of the alginate in the presence of CaCl_2_ [12]. As the CaCl_2_ diffused into the hydrogel and ionic crosslinks were formed the visual properties of the biomaterial ink changed. At 60 min, the CaCl_2_ had resulted in almost complete crosslinking of the biomaterial ink hydrogel.

**Figure 5:**
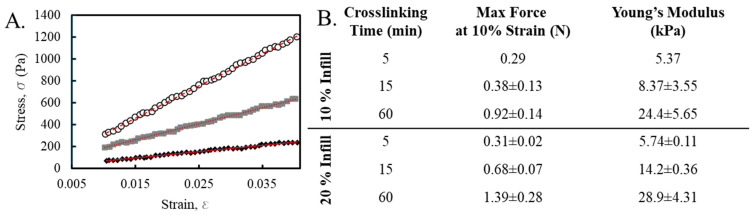
Mechanical testing of the printed bandage dressing squares. (**A**) Linear portion of example stress–strain curves for samples with 20% infill crosslinked for 5 min, R = 0.99 (Black Diamonds), 15 min, R = 0.99 (Grey Squares) or 60 min, R = 0.99 (Black Circles). The data were fit to linear regression, and the slope of the curve fit is Young’s modulus. (**B**) Maximum force at 10% strain and Young’s modulus of printed alginate/CMC hydrogel dressings after 5, 15, and 60 min of crosslinking in CaCl_2_.

Healthy human skin has Young’s modulus ranging from 5 kPa to 100 kPa when measured with indentation testing [49], and the hydrogel dressing should not exceed these values. Dressings exceeding the stiffness of normal skin may induce discomfort and secondary trauma [47,48]. Compression testing was used to calculate Young’s moduli for printed constructs with honeycomb infill patterns at densities of 10% and 20%. In addition, the effect of calcium-induced crosslinking time on the biomaterial ink stiffness was also determined (Figure 5). Stress and strain were calculated as described in the methods, and the slope of the linear portion of the stress/strain plots was used to calculate Young’s modulus (Figure 5B). The average moduli calculated from three prints ranged from 28.9 ± 4.31 kPa to 5.74 ± 0.11 kPa for the 20% infill when crosslinked for 60 min or 5 min in calcium chloride solution, respectively. In each case, the modulus increased with increasing length of time the printed dressing was exposed to CaCl_2_. The values are similar whether the infill was 10% or 20% as Young’s modulus measures the material properties and not necessarily the structure (e.g., the surface areas of the dressings printed with 10% or 20% infill are different and are part of the modulus calculation). The maximum force (N) detected in the 20% infill prints is generally higher than those detected in the prints with 10% infill. These data indicate that the crosslinked hydrogels have sufficient strength for use as a wound dressing. We chose to proceed with the 20% infill for all additional experiments.

### 3.4. Three-Dimensional Printing of EV-Laden Bio-Ink

**Figure 6:**
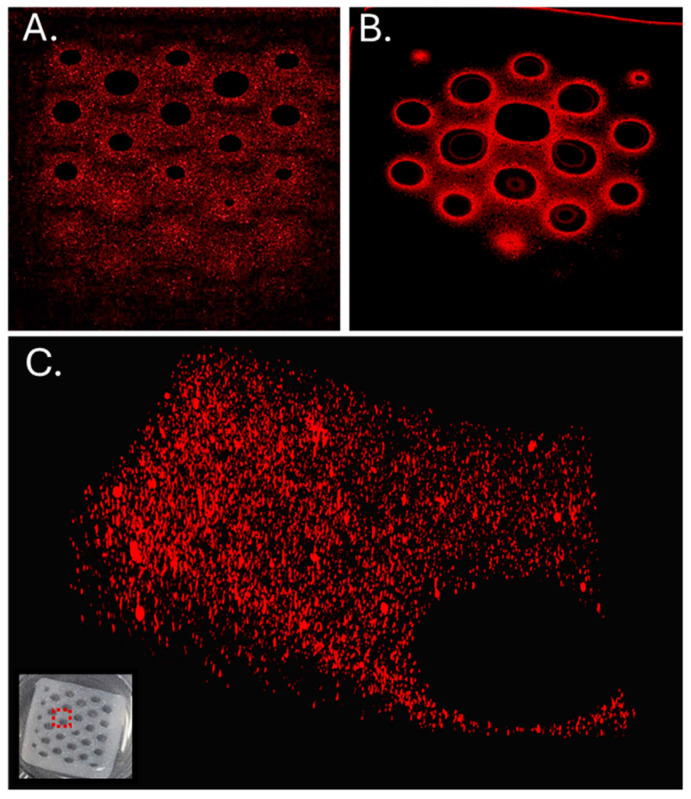
Bioprinted alginate/CMC dressing with reconstituted, red fluorescently labeled EVs. The bioactive dressing was bioprinted with 20% infill and imaged before crosslinking (**A**) and after crosslinking (**B**). A confocal microscopy z-stack, tile scan at 20X magnification visualized as a 3D projection of the red-boxed region of the alginate/CMC/EV dressing (inset) (**C**). The labeled EVs are distributed throughout the hydrogel dressing material.

Alginate/CMC bio-ink was mixed with red fluorescently labeled EVs, and the dressing (20 × 20 × 1 mm) was printed as described above and crosslinked with CaCl_2_ for 15 min (Figure 6). Healing of full-thickness skin wounds in mice improved with the injection of EVs directly into the wound [50]. Based on wound volume calculations, we incorporated a similar concentration of 0.5 µg of lyophilized EVs per µL wound volume. Fluorescent EVs were readily visible in both the non-crosslinked and crosslinked prints (Figure 6A,B), and EV uniform distribution was confirmed throughout the thickness of the hydrogel using confocal microscopy (Figure 6C). Thus, EVs can be combined successfully with the alginate/CMC bio-ink and 3D bioprinted into the shape of a dressing.

### 3.5. EV Release from 3D Printed Dressing

**Figure 7:**
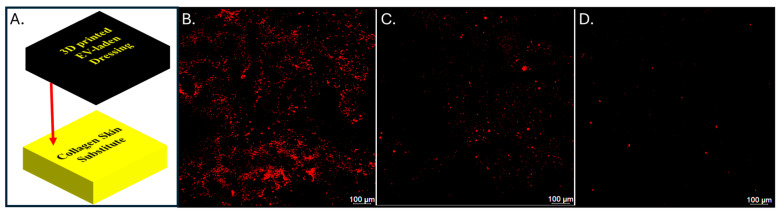
Transfer of EVs from dressing bio-ink to collagen blocks. (**A**) A 3D-printed collagen block was used to simulate skin to test the transfer of EVs from the printed dressing to the skin. The alginate/CMC dressing was printed using 20% infill, crosslinked, and cut to 10 mm × 10 mm × 3 mm. It was placed on top of a similar-sized collagen block and incubated at 37 °C in a 6-well plate with 1 mL of PBS to keep the collagen hydrated. (**B**) Positive control: a solution of fluorescently labeled EVs pipetted on top of the collagen and left to absorb. The collagen block was removed at 24 h and imaged for the appearance of labeled EVs transferred from the dressings that were crosslinked for (**C**) 10 min or (**D**) 60 min. Microscopy images taken at 20X magnification.

For maximum effectiveness of an EV-containing bioactive dressing in treating skin wounds, the EVs should be released from the dressing into the skin or wound surface, where they can exert their bioactivity. Because skin is composed predominantly of collagen, we simulated the use of the EV dressing on skin by placing the 3D bioprinted EV-laden dressing on top of a 3D printed square of methacrylated collagen type I (Figure 7). We evaluated the effect of crosslinking time on the transfer of EVs to the collagen. EVs were transferred to the surface of the collagen in 24 h more effectively when the alginate/CMC gel was crosslinked for a shorter time (e.g., 10 min vs. 60 min in CaCl_2_). These data serve as a demonstration that EVs can be incorporated into dressing using 3D printing and diffuse over time from the dressing to skin-type surfaces. Optimal conditions for alginate/CMC crosslinking times and the relation to EV transfer require further optimization and may be specific to the type of wound being treated.

### 3.6. Three-Dimensional Printing EV Dressing with a Ruggedized Printer

The laboratory bioprinter used for the development of this work is suitable for use in controlled laboratory environments and not designed for deployment in austere conditions. A custom ruggedized 3D printer has been specifically designed to be transported to and used in austere environments. This 3D printer has been built to military ruggedization standards and is capable of fused filament fabrication (FFF) 3D printing in addition to bioprinting and printing of conductive paste materials. The ruggedized 3D printer can operate from vehicle or marine batteries in addition to standard power to enable operation in unreliable or non-standard power scenarios common in conflict and disaster zones. Additionally, the ruggedization enables the 3D printer to be transported to and used in austere environments without damage. We have shown previously the ruggedized 3D printer transportation and use in austere desert and Arctic locations [19,20].

**Figure 8:**
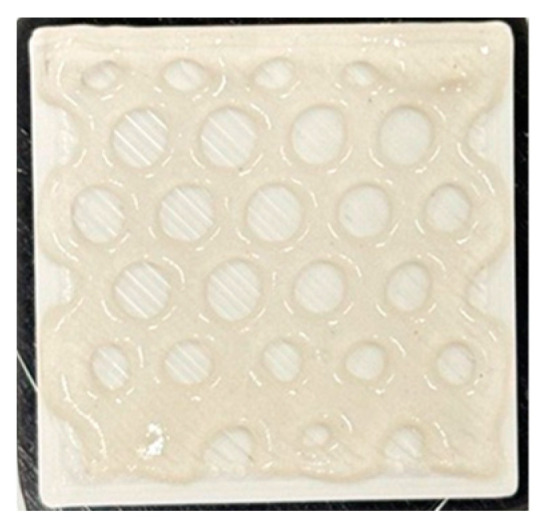
Three-dimensional printing and bioprinting of a wound dressing in a laboratory environment using a ruggedized 3D printer. The alginate/CMC dressing was bioprinted onto an FFF 3D printed PLA backing and crosslinked with CaCl_2_ solution for 10 min.

The bioactive dressing was first bioprinted using a ruggedized printer in the laboratory environment. To facilitate the application of the alginate/CMC EV-laden dressing onto a wound, a PLA thermoplastic backing was printed to transfer the hydrogel from the print bed to the wound. A 20 mm × 20 mm × 1 mm square design was used initially. The PLA backing was printed using a nozzle temperature of 210 °C and a bed temperature of 50 °C onto a glass microscope slide taped to the print bed using laboratory tape. The alginate/CMC biomaterial ink was dispensed using a pneumatic print nozzle with a 125 µm ceramic tip and 6–8 kPa pneumatic pressure. Like the laboratory printer tests, a honeycomb infill of 20% was used. The resulting dressing was then crosslinked for 10 min with CaCl_2_ solution by pipetting the solution on top of the dressing, followed by washing with PBS (Figure 8).

Although the same infill percentage and pattern were used, the shape fidelity and inner holes in the honeycomb pattern were improved over the commercial laboratory-grade printer due to the precision dispensing ability of the smart pump printheads used on the ruggedized printer.

**Figure 9:**
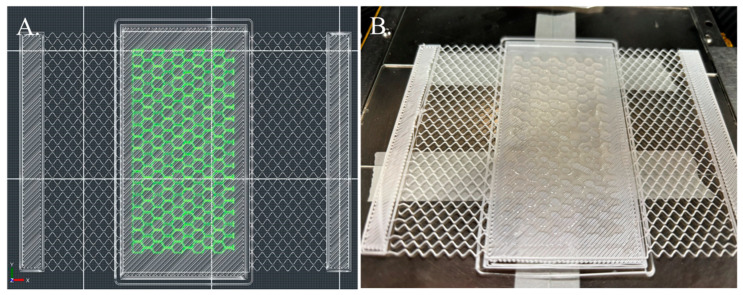
(**A**) CAD rendering and (**B**) bioprinted bandage with alginate/CMC EV-laden bio-ink dressing.

Next, a bandage design that incorporates a PLA backing, flexible mesh, and strips for adhesive on the sides was 3D printed. The EV-laden alginate/CMC dressing with a 1 mm height was bioprinted using the smart pump in the center of the bandage. Figure 9A depicts the 3D CAD renderings generated by the ruggedized printer slicing software, and Figure 9B shows the resulting print of a bandage with locations for the adhesive to be printed, the flexible mesh, and the backing and bioactive dressing.

### 3.7. Three-Dimensional Printing of a Bioactive Wound Dressing in an Austere Location

To demonstrate the feasibility of 3D printing hydrogel bandages containing EVs, the ruggedized 3D printer was deployed to a remote Arctic location. The logistics and feasibility of transporting the ruggedized printer and equipment to a point-of-need Arctic location during a military exercise were established [20]. Translation of advanced biotherapeutic technologies from the benchtop to the austere environments is becoming increasingly more important as medical evacuation is extended or improbable. This necessitates being able to manufacture biotherapeutics at the point of need in the absence of reliable cold-chain storage, logistics support, and diverse or interrupted electrical power scenarios.

EVs were transported to the austere Arctic location at room temperature in a lyophilized form. Lyophilized EVs can be stored for up to several months without detrimental impacts on bioactivity [17,18]. The stability of EVs in lyophilized form makes them an alternative to cell-based therapeutics in remote or austere locations. In these scenarios, limited cold-chain and cell-culture resources make translation of cell-based therapies difficult. Stable EVs represent an alternative that could enable the benefits of cell-based therapies to be realized in these locations.

**Figure 10:**
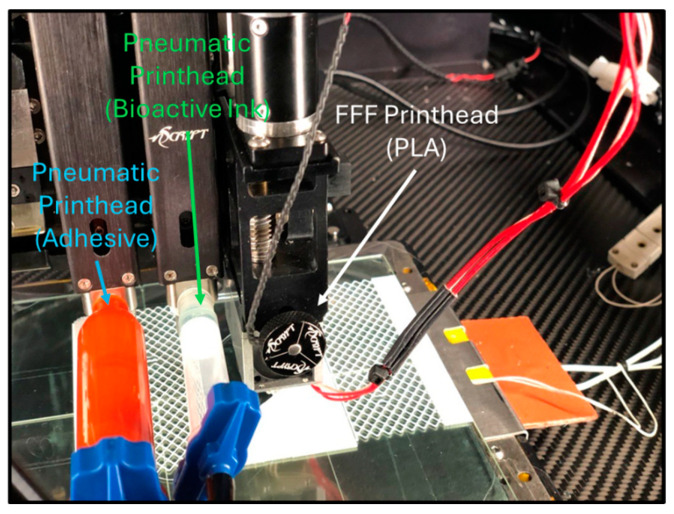
Ruggedized 3D printer for point-of-need manufacturing of a bioactive wound dressing. The 3D printer contained three printheads. A fused filament fabrication (FFF) printhead was used to print the PLA thermoplastic backing. Two pneumatic printheads were used to print the alginate/EV bioactive bio-ink and a commercially available adhesive.

EVs were reconstituted with PBS, mixed with alginate/CMC bio-ink in a syringe, and loaded onto the ruggedized printer (Figure 10), where the ink was used to print the same bandage shown in Figure 9. The bandages printed on the ruggedized printed in the lab setting and the austere environment were visually equivalent. While this work demonstrates the feasibility of the process and the value in operational and austere medicine, therapeutic applications [51,52,53,54] of EVs are not yet approved for human clinical use by the US Food and Drug Administration.

The success of EV-loaded hydrogels in wound treatment has been demonstrated with hydrogels including alginate, chitosan, polyurethane, and polyethylene glycol [7,55,56,57,58]. These publications report that EVs help full-thickness skin wound closing and re-epithelialization in rodent models. Thus, EVs are promising biological agents for wound healing therapy [59] that are shelf-stable and retain activity when lyophilized [17,18,28,29,30]. When combined with a hydrogel dressing, they make an ideal candidate for 3D bioprinting. Raw materials for alginate/CMC biomaterial ink can be stored long-term at room temperature. Once prepared, the ink remains stable at room temperature for a short to medium term and can be stored longer under controlled conditions. Lyophilized EVs are stable for up to 4 months at room temperature and longer at −20 °C [28]. In emergencies, these materials can be retrieved from storage and deployed with 3D printers when needed.

The demonstration of a 3D bioprinted EV-containing bandage shows the feasibility of point-of-need manufacturing and the promise of transporting and incorporating EVs into therapeutics in austere operating environments. This point-of-need manufacturing process is applicable to a wide range of needs, from wound care to the fabrication of replacement parts [20]. Three-dimensional printing has the advantage of permitting easily customizable bandage and dressing shapes and designs [60]. In addition, 3D printing is a fairly simple and rapid process to perform, and uses materials efficiently with little waste relative to other manufacturing methods. Thus, in resource-limited operational medicine scenarios, 3D printing is a promising solution and should be investigated in greater detail with more feasibility testing and measures of success so that it might be implemented as part of a standard medical response to disasters, conflicts, and in austere environments.

The 3D printed alginate/CMC dressing presented here is also easily modified to tune the stiffness of the crosslinked material such that EV release or transfer can be changed depending on the type of wound or healthcare need required. Moreover, the choice of material for the bandage backing is also variable. For example, in addition to PLA, polycaprolactone and other thermoplastics are useful for bandage backing [61,62]. Furthermore, the hydrogel can potentially be crosslinked with other antimicrobial cations such as Cu^2+^ and Zn^2+^ [63,64,65], or antimicrobial activity can be improved by blending with silver nanoparticles [66,67] or antibiotics.

There is a need for controlled experiments to compare and quantify outcomes of wound treatments at the point of need versus more conventional standards of care. Furthermore, a more detailed understanding of 3D-printed bioactive bandage design would come from pre-clinical trials using bandages with differing mechanical properties to treat a range of wounds, including burns, traumas, infections, and both chronic and acute skin wounds in animal models. Additional work is needed to optimize the therapeutic EV dose or concentration to promote effective wound healing and decrease scarification. The effects on wound healing and infection prevention or mitigation for different EV bandage properties will provide insights into optimal bandage construction for the promotion of wound healing to eventually enable the development of FDA-approved, clinically useful products and processes.

A coordinated effort between healthcare providers operating in austere environments with limited resources and laboratory research and development bioengineers is necessary to ensure the advanced therapeutics and 3D printing technologies developed for use in operational and austere scenarios meet the clinical and technical challenges unique to these environments. Additionally, regulatory approval and development of standards and certification of 3D printing equipment used in these environments are needed.

## 4. Conclusions

While there are still regulatory and optimization challenges to address, this work presents a potential solution to overcome the limitations in cold-chain storage and resources required to bring modern cell-based therapies to the battlefield or disaster ground by demonstrating the transportation and use of EVs derived from MSCs in an austere environment. A 3D bioprinted alginate/cellulose dressing capable of EV release was developed and characterized by rheology and compression testing. The alginate/cellulose bio-ink developed here can be stored at room temperature, and the printability was demonstrated on multiple 3D printers in the laboratory and on the ruggedized printer in an austere environment. The feasibility of point-of-need manufacturing an EV-laden hydrogel bandage for wound healing was demonstrated.

## Figures and Tables

**Table 1:** Results from mass spectrometry analysis of isolated EVs. Results confirmed the presence of key EV markers. All protein sequences are from humans and are identified with high confidence (FDR < 0.01) with LC-MS.

Group	Accession Code	Gene ID	Description (Alternative Name)	Coverage [%]	# Peptides
1. Transmembrane or GPI-anchored proteins associated with plasma membrane and/or endosomes	P05106	ITGB3	Integrin beta-3	13	10
	P31431	SDC4	Syndecan-4	13	2
	H7C1K4	SDC1	Syndecan-1 (Fragment)	8	1
	F8VWK8	CD63	Tetraspanin (Fragment)	8	2
	J3QRJ3	THY1	Thy-1 membrane glycoprotein (CD90)	10	1
2. Cytosolic proteins recovered in EVs	P07355	ANXA2	Annexin A2	59	15
	P11142	HSPA8	Heat shock cognate 71 kDa protein	45	28
	Q8WUM4	PDCD6IP	Programmed cell death 6-interacting protein (ALIX)	9	8
3. Major components of non-EV co-isolated structures	J3QTR3	RPS27A	Ubiquitin-40S ribosomal protein S27a (Fragment)	44	4
	M0QX76	RPS16	40S ribosomal protein S16 (Fragment)	20	1
	P02647	APOA1	Apolipoprotein A-I ^1^	17	3
	P04114	APOB	Apolipoprotein B-100 ^1^	3	12
	A0A087WWT3	ALB	Serum albumin ^1^	89	56
4. Transmembrane, lipid-bound, and soluble proteins associated with other intracellular compartments than PM/endosomes	P62805	H4C1	Histone H4	50	5
	Q5QNW6	H2BC18	Histone H2B type 2-F	36	5
	P10412	H1-4	Histone H1.4	23	5
	P07305	H1-0	Histone H1.0	16	3

^1^ Peptides were detected but were below the signal-to-noise threshold for quantitation. Coverage refers to how much of the protein sequence was determined, and # of peptides indicates how many peptides corresponding to the protein were resolved by mass spectrometry.

## Data Availability

The original contributions presented in the study are included in the article/supplementary material. Further inquiries can be directed to the corresponding author/s.

## Supplementary Materials

The following supporting information can be downloaded at https://www.mdpi.com/article/10.3390/bioengineering11080804/s1, Figure S1: Raw absorbance values at 280 nm of SEC column fractions collected from human MSC-conditioned media (MS-CCM).
