# Supplementary material for: An Exosome-Laden Hydrogel Wound Dressing That Can Be Point-of-Need Manufactured in Austere and Operational Environments
Source: Bioengineering (Basel). 2024 Aug 8;11(8):804. doi: 10.3390/bioengineering11080804 (PMC11351238; doi:10.3390/bioengineering11080804)
Supplement: Supplementary file 1 [file bioengineering-11-00804-s001.zip › bioengineering-3144045-supplementary.pdf]

Supplementary Information

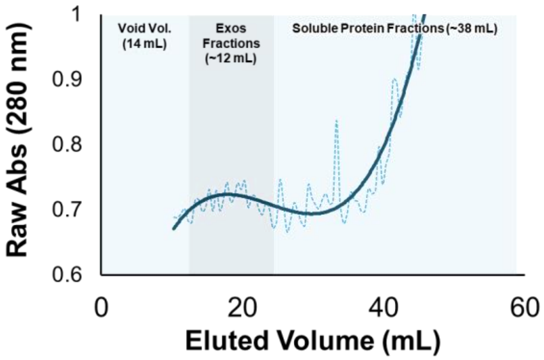

Supplementary Figure 1: Raw absorbance values at 280nm of SEC column fractions collected from human MSC conditioned media (MS-CCM). 1mL of MS-CCM was added to the column and fractions were collected in microcentrifuge tubes before being transferred to a 96 well plate for analysis on a microplate reader.
